# Supplementary material for: Transfer dynamics of multi-resistance plasmids in Escherichia coli isolated from meat
Source: PLoS One. 2022 Jul 7;17(7):e0270205. doi: 10.1371/journal.pone.0270205 (PMC9262221; doi:10.1371/journal.pone.0270205)

PLOS ONE

Transfer dynamics of multi-resistance plasmids in Escherichia coli isolated from meat

--Manuscript Draft--

**Manuscript Number:**	PONE-D-21-39054
**Article Type:**	Research Article
**Full Title:**	Transfer dynamics of multi-resistance plasmids in Escherichia coli isolated from meat
**Short Title:**	Plasmid transfer between E. coli strains
**Corresponding Author:**	Benno H. ter Kuile, Ph.D.<br>University of Amsterdam<br>Amsterdam, NETHERLANDS
**Keywords:**	Horizontal gene transfer; transfer rates; antimicrobial resistance; pilus morphology
**Abstract:**	Resistance plasmids are crucial for the transfer of antimicrobial resistance and thus form a matter of concern for veterinary and human healthcare. To study plasmid transfer, foodborne Escherichia coli isolates harboring one to five known plasmids were co-incubated with a general recipient strain. Plasmid transfer rates under standardized conditions varied by a factor of almost 106, depending on the recipient/donor strain combination. After 1 hour transconjugants never accounted for more than 3% of the total number of cells. Transconjugants were formed from 14 donors within 1 hour of co-incubation, but in the case of 3 donors 24 hours was needed. Transfer rates were also measured during longer co-incubation, between different species and during repeated back and forth transfer. Longer co-incubation resulted in the transfer of more types of resistance. Maximum growth rates of the donor strains varied by a factor of 3. Donor strains often had higher growth rates than the corresponding transconjugants, which grew at the same rate as or slightly faster than the recipient. Hence, possessing one or more plasmids does not seem to burden the harboring strain metabolically. Transfer was species specific and repeated transfer of one plasmid did not result in different transfer rates over time. Transmission Electron microcopy was used to analyze the morphology of the connection between co-incubated strains. Connection by more pili between the cells resulted in better aggregate formation and corresponded with higher transfer rates.
**Order of Authors:**	Tania Sabina Darphorn, Ph.D.
	Belinda Koenders-Van Sint Anneland
	Grootemaat Anita
	Nicole Van der Wel
	Stanley Brul
	Benno H. ter Kuile, Ph.D.
**Additional Information:**	
**Question**	**Response**
**Financial Disclosure**<br><br>Enter a financial disclosure statement that describes the sources of funding for the work included in this submission. Review the submission guidelines for detailed requirements. View published research articles from *PLOS ONE* for specific examples.<br><br>This statement is required for submission and **will appear in the published article** if	no

Powered by Editorial Manager® and ProduXion Manager® from Aries Systems Corporation

the submission is accepted. Please make sure it is accurate.<br><br>**Unfunded studies**<br>Enter: *The author(s) received no specific funding for this work.*<br><br>**Funded studies**<br>Enter a statement with the following details:<br>• Initials of the authors who received each award<br>• Grant numbers awarded to each author<br>• The full name of each funder<br>• URL of each funder website<br>• Did the sponsors or funders play any role in the study design, data collection and analysis, decision to publish, or preparation of the manuscript?<br>• **NO** - Include this sentence at the end of your statement: *The funders had no role in study design, data collection and analysis, decision to publish, or preparation of the manuscript.*<br>• **YES** - Specify the role(s) played.<br><br>* typeset	
**Competing Interests**<br><br>Use the instructions below to enter a competing interest statement for this submission. On behalf of all authors, disclose any competing interests that could be perceived to bias this work—acknowledging all financial support and any other relevant financial or non-financial competing interests.<br><br>This statement is required for submission and **will appear in the published article** if the submission is accepted. Please make sure it is accurate and that any funding sources listed in your Funding Information later in the submission form are also declared in your Financial Disclosure statement.<br><br>View published research articles from *PLOS ONE* for specific examples.	The authors have declared that no competing interests exist

**NO authors have competing interests**<br><br>Enter: *The authors have declared that no competing interests exist.*<br><br>**Authors with competing interests**<br><br>Enter competing interest details beginning with this statement:<br><br>*I have read the journal's policy and the authors of this manuscript have the following competing interests: [insert competing interests here]*<br><br>* typeset	
**Ethics Statement**<br><br>Enter an ethics statement for this submission. This statement is required if the study involved:<br><br>• Human participants<br>• Human specimens or tissue<br>• Vertebrate animals or cephalopods<br>• Vertebrate embryos or tissues<br>• Field research<br><br>Write "N/A" if the submission does not require an ethics statement.<br><br>General guidance is provided below. Consult the submission guidelines for detailed instructions. **Make sure that all information entered here is included in the Methods section of the manuscript.**	Not relevant

**Format for specific study types**<br><br>**Human Subject Research (involving human participants and/or tissue)**<br>• Give the name of the institutional review board or ethics committee that approved the study<br>• Include the approval number and/or a statement indicating approval of this research<br>• Indicate the form of consent obtained (written/oral) or the reason that consent was not obtained (e.g. the data were analyzed anonymously)<br><br>**Animal Research (involving vertebrate animals, embryos or tissues)**<br>• Provide the name of the Institutional Animal Care and Use Committee (IACUC) or other relevant ethics board that reviewed the study protocol, and indicate whether they approved this research or granted a formal waiver of ethical approval<br>• Include an approval number if one was obtained<br>• If the study involved *non-human primates*, add *additional details* about animal welfare and steps taken to ameliorate suffering<br>• If anesthesia, euthanasia, or any kind of animal sacrifice is part of the study, include briefly which substances and/or methods were applied<br><br>**Field Research**<br><br>Include the following details if this study involves the collection of plant, animal, or other materials from a natural setting:<br>• Field permit number<br>• Name of the institution or relevant body that granted permission	
**Data Availability**<br><br>Authors are required to make all data underlying the findings described fully available, without restriction, and from the time of publication. PLOS allows rare exceptions to address legal and ethical concerns. See the PLOS Data Policy and FAQ for detailed information.	Yes - all data are fully available without restriction

A Data Availability Statement describing where the data can be found is required at submission. Your answers to this question constitute the Data Availability Statement and **will be published in the article**, if accepted.<br><br>**Important:** Stating 'data available on request from the author' is not sufficient. If your data are only available upon request, select 'No' for the first question and explain your exceptional situation in the text box.<br><br>Do the authors confirm that all data underlying the findings described in their manuscript are fully available without restriction?	
**Describe where the data may be found in full sentences. If you are copying our sample text, replace any instances of XXX with the appropriate details.**<br><br>• If the data are **held or will be held in a public repository**, include URLs, accession numbers or DOIs. If this information will only be available after acceptance, indicate this by ticking the box below. For example: *All XXX files are available from the XXX database (accession number(s) XXX, XXX.).*<br>• If the data are all contained **within the manuscript and/or Supporting Information files**, enter the following: *All relevant data are within the manuscript and its Supporting Information files.*<br>• If neither of these applies but you are able to provide **details of access elsewhere**, with or without limitations, please do so. For example:<br><br>*Data cannot be shared publicly because of [XXX]. Data are available from the XXX Institutional Data Access / Ethics Committee (contact via XXX) for researchers who meet the criteria for access to confidential data.*<br><br>*The data underlying the results presented in the study are available from (include the name of the third party*	All relevant data are within the manuscript and its Supporting Information files

and contact information or URL).<br>• This text is appropriate if the data are owned by a third party and authors do not have permission to share the data.<br><br>* typeset	
Additional data availability information:	

manuscript

Click here to access/download;Manuscript;Darphorn et al. transfer 12-2021.docx

Transfer dynamics of multi-resistance plasmids in *Escherichia coli* isolated from meat

Tania S. Darphorn[1], Belinda B. Koenders-van Sintanneland[1], Anita E. Grootemaat[2], Nicole N. van der Wel[2], Stanley Brul[1], Benno H. ter Kuile[1,3 *]

[1]Laboratory for Molecular Biology and Microbial Food Safety, Swammerdam Institute for Life Sciences, University of Amsterdam, Science Park 904, 1098 XH Amsterdam, The Netherlands.
[2]Department of Cell Biology and Histology, Electron Microscopy Centre Amsterdam, Academic Medical Center, Amsterdam, The Netherlands.
[3]Netherlands Food and Consumer Product Safety Authority, Office for Risk Assessment, Utrecht, The Netherlands.

* Author for correspondence
E-mail: b.h.terkuile@uva.nl ; phone: +31-6-46596684

Short title: Transfer of resistance plasmids between E. coli strains

Keywords: Horizontal gene transfer ; transfer rates ; antimicrobial resistance; pilus morphology

Abstract

Resistance plasmids are crucial for the transfer of antimicrobial resistance and thus form a matter of concern for veterinary and human healthcare. To study plasmid transfer, foodborne *Escherichia coli* isolates harboring one to five known plasmids were co-incubated with a general recipient strain. Plasmid transfer rates under standardized conditions varied by a factor of almost 10^6, depending on the recipient/donor strain combination. After 1 hour transconjugants never accounted for more than 3% of the total number of cells. Transconjugants were formed from 14 donors within 1 hour of co-incubation, but in the case
were
of 3 donors 24 hours was needed. Transfer rates were also measured during longer co-incubation, between different species and during repeated back and forth transfer. Longer co-incubation resulted in the transfer of more types of resistance. Maximum growth rates of
DELETE the
the donor strains varied by a factor of 3. Donor strains often had higher growth rates than the corresponding transconjugants, which grew at the same rate as or slightly faster than the recipient. Hence, possessing one or more plasmids does not seem to burden the harboring strain metabolically. Transfer was species specific and repeated transfer of one plasmid did not result in different transfer rates over time. Transmission Electron microcopy was used to analyze the morphology of the connection between co-incubated strains. Connection by more pili between the cells resulted in better aggregate formation and corresponded with higher transfer rates.

Introduction

Resistance plasmids play a crucial rule in the spread of antimicrobial resistance in veterinary and human healthcare (1-4). *Escherichia coli* infections are becoming increasingly untreatable due to the encoded resistance genes on plasmids such as extended spectrum beta-lactamases (ESBL), beta-lactamases (BL) and tetracycline resistance, especially observed in livestock (5-8). Pathogens resistant to antimicrobials can transfer from animals directly to farmers (9, 10) or from foodstuffs to human consumers human healthcare (11). All application s of antibiotics in farming and human healthcare can increase the spread of resistance plasmids and genes (12). Plasmids are able not only to spread between cells of the same species but also to different species of bacteria (13-18). To better understand the spreading of resistance mediated by plasmids, we explored the dynamics of conjugation and plasmid transfer.

The conjugation machinery encoded on plasmids enable enables them to transfer from cell to cell (19-21), but conjugation has been shown to be mostly species specific (14, 16). Conjugation rates often remain low as the process has a high metabolic cost (22). There are multiple conjugative systems, which have been used to characterize plasmid types in combination with their incompatibility groups (18). The incompatibility groups IncI, IncF and IncX all have their own type of conjugative system that transfers plasmids at different rates (13, 18, 19, 23).

Resistant *E. coli* strains isolated from meat destined for the consumer market often contain multiple plasmids in a single strain (24). These plasmids consisted of several types, but the IncI, IncX and IncF types were most present in these isolates. The plasmids possessed a wide variety of resistance genes between them. This raises several questions: what effect does the cellular presence of multiple plasmids have on conjugation? Is transfer with cells harboring multiple plasmids affected by length of the co-incubation or co-incubation with different species? A final question is whether continuous back and forth transfer of one such naturally occurring plasmid influences the efficiency of the transfer process.

To answer these questions, a standard transfer method was designed to measure actual transfer events while excluding the effects of competition and growth. A set of donor *E. coli*

isolated from foodstuffs, is co-incubated with a general recipient to obtain transconjugants. Transfer rates are correlated to the growth rates of the donor, recipient and transconjugant strains. The transfer method was adapted to also include longer co-incubation, different species and continuous transfer as variables. Lastly, transmission electron microscopy was used to examine the morphology of pili between the co-incubated strains.

Materials & Methods

Bacterial strains, solutions, MICs and growth rate. All strains used are shown in table 1. The *Escherichia coli* donor strains were isolated from foodstuffs by the Dutch Food and Consumer Product Safety Authority (NVWA) and characterized by Wageningen Bioveterinary research (WBVR). Dr. Kees Veldman of WBVR selected and donated 28 strains that were known to contain plasmids and that originated from turkey, bovine or chicken meat. Out of this set 17 selected for having at least beta-lactam resistance were used in this study. The NVWA originally established the resistance with a sensitivity test (25) and this was confirmed afterwards using MIC assays (26). Plasmid presence was initially confirmed by the detection of incompatibility groups (27) and later confirmed by plasmid isolation and long and short read sequencing (24). These sequences were analyzed using CGEs PlasmidFinder 2.1 (28) and ResFinder 4.0 (29) to determine and confirm and find other incompatibility groups and resistance genes. The chloramphenicol resistant ($chlor^R$) *E. coli* MG1655 YFP (kindly provided by MB Elowitz (30)) was used as common recipient strain in transfer experiments. Another *E. coli* MG1655 strain was evolved to obtain enrofloxacin resistance by stepwise increasing the antibiotic concentration. This strain was used as a second standard recipient whenever the donor strain already expressed chloramphenicol resistance. Recipients were chosen so that the donor could not grow on the plate selecting for the transconjugant. Another *E. coli* strain was used for continuous back and forth transfer experiments: *E. coli* JW3686. This strain is an indole knockout mutant with inserted kanamycin resistance (kan^R). Three other organisms

were used as recipient strains in experiments to examine species specificity of the transfer process: *Pseudomonas aeruginosa* POA-1 and PA-1, as well as *Enterococcus faecalis* OG1RF

Defined minimal mineral medium containing 55 mM glucose with a pH of 6.9 and a buffer of 15.6 g/L Na2H2PO4 (31) was used to grow strains overnight at 37°C degrees and shaken at 200 rpm. The same minimal medium without glucose was used for standard transfer experiments to prevent confounding of the outcome by growth. Selective plates were made with Luria broth (LB) (1% NaCl; 0.5% yeast extract; 1% bactotryptone) with 2% agar and appropriate antibiotic stocks. Stock solutions of 10 mg/ml antibiotics for ampicillin, chloramphenicol, enrofloxacin, kanamycin and tetracycline were filter sterilized and stored at 4°C for up to 2 weeks maximum. The final concentration of antibiotics in the selective plates were set to 64 µg/ml.

MIC and growth rates of the donor, recipient and transconjugant strains were measured as described by Schuurmans, Nuri Hayali (26) in 96-well plates in a ThermoScientific Multiskan FC spectrophotometer plate reader. Plates were shaken and kept at 37 °C in a final volume of 150 µl with a starting OD595 of 0.05 for 23 hours. Antibiotic concentrations increasing by a factor of 2 and ranging from 1 µg/ml to 2048 µg/ml were used. The lowest concentrations that limited final OD to 0.2 or less was reported as MIC. The maximum specific growth rate (µmax) was determined out of the growth curves that were obtained from 8 wells with minimal medium. This method was used for growth rate determination, because of the high reproducibility.

Transfer experiments. For most transfer experiments a standard method was established to reduce the influence of other parameters, such as growth and increase reproducibility. Cells of an overnight culture were spun down at 4400 rpm for 15 minutes, the old medium was is removed and replaced with minimal medium without glucose. After careful mixing, the cells were are starved for 4 hours at 37 °C and shaken at 200 rpm. To start the transfer experiment, the starved donor and recipient strains were mixed at a in a 1:1 ratio with a final OD600 of 0.25 in minimal medium without glucose. Preliminary experiments showed that after 1 and 24 hours of co-incubation the total cell number remained stable and that the cells were still viable. Total numbers of recipient, donor and transconjugant cells were determined by plating

dilutions on appropriate selective LB agar plates. Plates to select for transconjugants
were
contained both antibiotics that the donor and recipient strain respectively are resistant to.
s by MIC assay and later also by WHICH GENE??
Strains were confirmed as transconjugant with MIC assays and later also DNA sequencing (Genbank accession numbers MW390511 to MW390552). 2 or 3 transconjugants were isolated from the selective plates and tested for MIC and growth rate and stored in glycerol stocks at -80 °C. In some experiments, this standard method was adapted according to the experimental design. All transfer experiments were performed in triplicate.

E. coli 3170 was attempted to mate with other species (*P. aeruginosa* and *E. faecalis*) in multiple transfer experiments. Apart from the standard method, a second method was used with minimal medium with added glucose and a third method with the use of LB medium instead of minimal medium for 1 and 24 hours. Other than the difference in medium the set-up of the experiment stayed the same. In an additional experiment, a 1:1 mixed solution of recipient and donor strains with an OD600 of 1 in an Eppendorf tube was directly plated on selective plates.

One plasmid was transferred multiple times back and forth between 2 recipient strains. A transconjugant containing the IncI1 plasmid of *E. coli* 3170 (*E. coli* MG1655 pIncI3170) was mated with the standard recipient using the standard method and with *E. coli* JW3686 (kan^R) with glucose in the medium as this recipient otherwise did not survive the experiment. To continue the transfer of the plasmid, a transconjugant was isolated and grown overnight from the 1-hour co-incubation plates and used as the new donor strain (*E. coli* JW3686 pIncI3170) in a new mating with *E. coli* MG1655 (chlor^R) as recipient. In the single case that the 1-hour plates did not yield any transconjugants, a transconjugant from the 24-hour plates was isolated instead. These steps were repeated 11 to 12 times. Transconjugant strains were tested for MIC and indole to ascertain that they were true transconjugants. The indole test discriminated the indole knockout strain *E. coli* JW3686 from *E. coli* MG1655, as the former turns yellow when mixed with Kovac's reagent while the latter will color pink for the presence of indole.

Transformation. Transformations were attempted by mixing competent *E. coli* MG1655 with either plasmid DNA or dead cells from *E. coli* 3153 and 2073. Plasmid DNA was isolated using

the Qiagen Plasmid Maxi Kit. Dead cells were obtained by pasteurizing cells at 72 °C for 15 seconds. The transformation is done by heat shock at 42 °C for 45 seconds followed by cold shock on ice for a maximum of 5 minutes for plasmid DNA and 30 minutes for dead cells. Before plating the transformation on selective plates, the cells are recovered in S.O.C. medium (2% Tryptone; 0,5% Yeast Extract; 0,4% glucose; 10 mM NaCl; 2,5 mM KCl; 10 mM $MgCl_2$) for 60 minutes for plasmid DNA and 2 or 5 hours for dead cells at 37 °C and 200 rpm. The dead cells were additionally transformed without the heat and cold shocks. Transformations with water and a control plasmid (pUC19) were performed as a negative and positive control, respectively.

Transmission electron microscopy Pilli of *E. coli* 3153, 2073, MG1655 and JW3686 were visualized using transmission electron microscopy (TEM). Part of the fresh overnight cultured cells were fixed with 4% paraformaldehyde (PFA) and allowed to slowly settle for 4 hours and transported and stored at 4 °C. Just before EM visualization fixed and unfixed cells were contrasted on a copper formvar coated grid using uranyl acetate. Fresh non-fixed cells showed a better result than PFA fixed cells as these did not show as much or any pili. Fresh cells were therefore used for the final analysis. Both single and mixed populations were visualized using TEM. The mixed populations consist of a 1:1 ratio of a donor and recipient strain and were co-incubated for no longer than 2 hours before storing and transportation at 4 °C. Visualization and handling of the TEM (Tecnai T12 at 120 kV with a Veleta digital camera) was performed at the Electron Microscopy Centre Amsterdam (EMCA, Amsterdam UMC).

Results

Transfer experiments were conducted to determine the rates at which transconjugants can be formed when two potential mates are co-incubated together. The mating procedure was designed to eliminate the effect of growth and competition, so that the numbers correspond to actual transfer events. Transfer rates ranged between 0,2 to approximately 30.000

corresponded
transconjugants per million cells per hour (Fig. 1). These numbers correspond at maximum to 3% of the total amount of cells. Three donor strains were never able to produce transconjugants after 1 hour but did form them after 24 hours. One strain did not form any
transconjugant at all
transconjugants at all (not shown in the graphs). Two of the three strains that transferred resistance only after 24 hours contained only a single plasmid, which belonged to the IncF
was contained
type. Strain 2073 is the exception which contains both a IncI and IncX4-type plasmids, but still
contained
transferred at a low rate. All other strains that contain an IncI-like plasmid transferred
varied
plasmids within 1 hour, but the transfer rates vary highly with no discernable trend. As control experiments, some transfers were performed with different selective plates, using tetracycline instead of ampicillin, to see if tetracycline harboring plasmids could also be selected for. Standard methods and methods with LB and added glucose did not yield any transconjugants for the tested donor strains during 1 hour or 24 hour incubations.

Transformation was performed as a control experiment with two strains to determine whether transformation of large plasmids was possible, thus assessing a possible role of transformation as a factor influencing transfer rates. Transformation for both strains 3153 (fast transfer) and 2073 (slow transfer) showed no plasmid uptake using both dead cells or pDNA in different media (LB and SOC). A control plasmid (pUC19) was able to be transformed into the competent cells.

Possessing plasmids is supposed to have a metabolic burden, lowering the growth rate. The
ranged from
maximum specific growth rates of the donor strains vary from 0,4 to 1,7 h^{-1} (Fig. 2). The
equaled the least to the recipient cells
growth rates of the transconjugants vary far less and equal at least that of the recipient cell
exceeded suggested
(0,42 h^{-1}), but mostly exceed it by a factor of maximally 1.6. This observation suggests that the metabolic burden was not pertinent in these transconjugants.

Transfer between widely different species was examined using *Pseudomonas aeruginosa* and *Enterococcus faecalis* as recipients and *E. coli* ESBL 3170 as donor. Strain ESBL 3170 was selected
contained were
because it contains plasmid types that are found in a wide variety of bacterial species. Multiple attempts under different conditions did not yield any transconjugants (Table 2). The methods of transfer attempted were the standard method, addition of glucose, using LB medium instead of Evans minimal medium and direct plating in high concentrations as well

as using longer incubation times. The donor strain had an average transfer rate when co-incubated with the standard *E. coli* recipient.

To examine whether the transfer rate would increase after repeated transconjugation because the plasmid could would adapt to it, a transfer experiment was conducted consisting of repeated back and forth transfer of one specific plasmid. Contrary to our expectation this did not influence transfer rate nor or resistance. These transfers were performed using the standard method (Fig. 3A) and were repeated with the addition of glucose (Fig. 3B) for 12 or 11 consecutive transfers, respectively. The transconjugants' MICs for ampicillin, kanamycin and chloramphenicol did not change throughout the transfers. This outcome suggests that the combination of donor and recipient determined the determines transfer efficiency.

Many donor strains harbor more than one plasmid, see Table 1. If a donor strain harbors several plasmids, multiple or different plasmids could be transferred at a time. To see whether there was any difference in transferred resistance all transconjugants from transfer with donor strain 2082 that harbored harbors 5 plasmids were isolated and tested for the MIC for ampicillin, chloramphenicol (Fig. 4A), tetracycline (Fig. 4B) and kanamycin (Fig. 4C). The resistance for both chloramphenicol and ampicillin stayed similar over 24 hours as expected because of the selective plates. Higher resistance levels were are reached for both tetracycline and kanamycin after 24 hours co-incubation compared to after 1 hour. This suggested suggests that more plasmids were transferred during prolonged co-incubation.

Electron microscopy. TEM images were made from of cells to determine whether if any structural differences could be observed between a slow donor, a fast donor and the recipient strain. Two donor strains were compared: *E. coli* 2073, because it transferred only after 24 hours, and *E. coli* 3153 which transfered faster. transfers quickly. Similarities were detected in shape and size of the cells, as well as the presence of a single flagellum. Differences between the two strains were detected in the number of pili and the formation of cell-aggregates. *E. coli* 2073 showed some small pili (fig. 5A) and formed some small aggregates (data not shown) whereas *E. coli* 3153 showed more pili (fig. 5B). *E. coli* MG1655 carried several contained many flagella per cell (fig. 5C), where *E. coli* JW3686 contained only 1 or no flagellum (fig. 5D). Aggregates of *E. coli* 3153 showed contained more cells, both alone and in co-incubation with *E. coli* MG1655 (fig. 5E) and cells were are covered

by many pili. The pili, but also some flagella, seem to connect to a neighbouring cells' membrane, possibly enabling transfer of plasmids. As these cells are dried and thus flattened out on the formvar film coating on the EM grid, it is impossible to confirm actual fusion between the pili or flagella with the recipient cell. However, as multiple pili and some flagella are bridging the space between two cells, it suggests transfer of plasmids.

DISCUSS: Was a flagella ever before reported to transfer plasmids???

Discussion

In order to ensure that the experiments are comparable to each other the effect of growth during the incubations was eliminated by the absence of a carbon and energy source. As a result the absolute numbers of plasmid transfers in this study are comparatively low. Newly formed transconjugants never surpass more than 3% of the whole population after one hour of co-incubation. The role of energy stems from the metabolic costs of expression of the conjugative machinery. Continuous expression is adversary for the hosts survival (22). Thus, plasmids have adapted to keep conjugative transfer low and only some cells in the population get triggered to initiate transfer when they might be in the presence of a plasmid free host (32, 33). Still, plasmid transfer usually seems to be initiated quickly, within 1 hour of co-incubation as observed in 14 out of 17 cases. However, some strains take longer before successful transfer is established. Possibly the rate of transfer depends on the ability to form pili, and indeed, the electron micrographs demonstrated presence of pili at the time points investigated.

were
was adverse for
survival of the host ...
was
depended
investigated time ..

Transfer rates are affected by the number of plasmids a cell possesses, as all strains with two or more plasmids can transfer quickly, while two strains with one plasmid transfer slower. Increased conjugative transfer in the presence of other plasmids in the cell delays plasmid extinction (34). So, multiple plasmids in one cell could increase plasmid transfer rates to survive. The two strains having only one plasmid contained an IncF plasmid. The IncF plasmids are on average larger in size than other plasmids found in this dataset, 130 kb compared to

would be

90-120 kb for IncI plasmids and 30-60 kb for IncX plasmids (24). IncF plasmids have an approximately 400 times lower conjugation rate than IncX plasmids, but a marginally higher transfer rate of 2.5 times than that of IncI plasmids (13). Conjugation rates of IncF plasmids have been studied more often than those of IncX and IncI plasmids. More hours of co-
in the transfer of additional or different plasmids ..
incubation result in transfer an additional or different plasmid because the cells are longer in contact with each other. The rate for taking up a second plasmid was in some cases increased when compared to the transfer rate of the first plasmid (35).

Plasmid transfer was confirmed to be species specific as attempts to obtain transconjugants with other gram-negative or gram-positive bacteria as recipients were unsuccessful. This is in
plasmid
line with other studies demonstrating that plasmids transfer happens more to kin and closely
strains
related species (14, 16). However, this only seems to hold true for liquid mating rather than filter mating (13).

Growth rates of donor strains differed strongly, even though they were all *E. coli* strains. The newly obtained plasmids do not produce a metabolic burden to *E. coli* MG1655, because the acquisition of one or more plasmids does not reduce the growth rate. This indicates that these transconjugants can compete well with the recipient strain but would still be outcompeted in most cases (13 out of 17) by the donor strain population. In 4 out of 17 cases, the transconjugant strain would become the dominant population. A similar pattern was observed in *E. coli* harboring ESBL-plasmids (36). In that study most plasmids did not reduce the host growth rate significantly (8 out of 15). Only in some cases they grew less (4 out of 14) or more (2 out of 14). However, plasmids can burden the host just after the transfer event, before adapting over the first 24 hours towards a stable growth rate (37). The plasmids thus effectively reduce their costs in a new host within the first 24 hours in order not to be outcompeted (32, 33, 36).

The comparison of the morphology of slow and fast mating cells using TEM suggests that aggregate formation and number of pili influence transfer rates. Pili are well known to have a role in the formation of aggregates. Aggregates and biofilms are hotspots for plasmid transfer (38-42), because increased cell-to-cell contact results in a higher chance of transfer
contained
events. The two donor strains compared in this study contain two similar plasmids, but one

contained

contains an extra IncF plasmid. IncF plasmids can stimulate the formation of biofilms, which is after all a type of aggregate formation (43, 44). Possibly the extra IncF plasmid in *E. coli*

DELETE the ... enabling a better

3153 produces the many pili, enabling the better formation of aggregates.

Acknowledgements

strains

We thank Kees Veldman and Ben Wit for providing the resistant strain and stimulating discussions. The students Mireille Nulkes, Siem de Haan, Veronica Zampieri, Ivan Fung, Eline de Waard, Sidra Kashif, Selin Alpagot, Eva Kozanli and Hannah Boersma performed transfer experiments, transformations, MIC, growth rate assays and TEM visualization as part of their degree or internship.

Author Contributions

TD and BtK conceived the project. SB assisted in the design of experiments. TD and BKvS performed most experiments. NvdW and AG performed TEM imaging and created the TEM image figure 5. TD performed analysis of the data. TD and BtK wrote the manuscript. All authors critically reviewed the manuscript and approved the final version.

Competing interest

The authors have no competing interests to declare.

References

1. Carattoli A. Plasmids and the spread of resistance. Int J Med Microbiol. 2013;303(6-7):298-304.
2. Levy SB, Marshall B. Antibacterial resistance worldwide: causes, challenges and responses. Nat Med. 2004;10(12 Suppl):S122-9.
3. Lopatkin AJ, Meredith HR, Srimani JK, Pfeiffer C, Durrett R, You L. Persistence and reversal of plasmid-mediated antibiotic resistance. Nat Commun. 2017;8(1):1689.
4. Sommer MOA, Munck C, Toft-Kehler RV, Andersson DI. Prediction of antibiotic resistance: time for a new preclinical paradigm? Nat Rev Microbiol. 2017;15(11):689-96.
5. Kaesbohrer A, Bakran-Lebl K, Irrgang A, Fischer J, Kampf P, Schiffmann A, et al. Diversity in prevalence and characteristics of ESBL/pAmpC producing E. coli in food in Germany. Vet Microbiol. 2019;233:52-60.
6. Stine OC, Johnson JA, Keefer-Norris A, Perry KL, Tigno J, Qaiyumi S, et al. Widespread distribution of tetracycline resistance genes in a confined animal feeding facility. Int J Antimicrob Agents. 2007;29(3):348-52.
7. Tacconelli E, Carrara E, Savoldi A, Harbarth S, Mendelson M, Monnet DL, et al. Discovery, research, and development of new antibiotics: the WHO priority list of antibiotic-resistant bacteria and tuberculosis. The Lancet Infectious Diseases. 2018;18(3):318-27.
8. Verraes C, Van Boxstael S, Van Meervenne E, Van Coillie E, Butaye P, Catry B, et al. Antimicrobial resistance in the food chain: a review. Int J Environ Res Public Health. 2013;10(7):2643-69.
9. Dierikx C, van der Goot J, Fabri T, van Essen-Zandbergen A, Smith H, Mevius D. Extended-spectrum-beta-lactamase- and AmpC-beta-lactamase-producing Escherichia coli in Dutch broilers and broiler farmers. J Antimicrob Chemother. 2013;68(1):60-7.
10. Fischer J, Hille K, Ruddat I, Mellmann A, Köck R, Kreienbrock L. Simultaneous occurrence of MRSA and ESBL-producing Enterobacteriaceae on pig farms and in nasal and stool samples from farmers. Veterinary Microbiology. 2017;200:107-13.
11. Mughini-Gras L, Dorado-García A, van Duijkeren E, van den Bunt G, Dierikx CM, Bonten MJM, et al. Attributable sources of community-acquired carriage of Escherichia coli containing β-lactam antibiotic resistance genes: a population-based modelling study. The Lancet Planetary Health. 2019;3(8):e357-e69.
12. CDC. Antimicrobial Resistance, Food and Food Animals 2021 [updated June 14, 2021. Available from: https://www.cdc.gov/drugresistance/food.html.
13. Alderliesten JB, Duxbury SJN, Zwart MP, de Visser J, Stegeman A, Fischer EAJ. Effect of donor-recipient relatedness on the plasmid conjugation frequency: a meta-analysis. BMC Microbiol. 2020;20(1):135.
14. Dimitriu T, Marchant L, Buckling A, Raymond B. Bacteria from natural populations transfer plasmids mostly towards their kin. Proc Biol Sci. 2019;286(1905):20191110.
15. Dionisio F, Matic I, Radman M, Rodrigues OR, Taddei F. Plasmids spread very fast in heterogeneous bacterial communities. Genetics. 2002;162:1525-32.
16. Sakuda A, Suzuki-Minakuchi C, Okada K, Nojiri H. Conjugative Selectivity of Plasmids Is Affected by Coexisting Recipient Candidates. mSphere. 2018;3(6).
17. Threlfall EJ, Ward LR, Frost LS, Willshaw GA. The emergence and spread of antibiotic resistance in food-borne bacteria. International Journal of Food Microbiology. 2000;62:1-5.

18. Grohmann E, Muth G, Espinosa M. Conjugative plasmid transfer in gram-positive bacteria. Microbiol Mol Biol Rev. 2003;67(2):277-301, table of contents.
19. Arutyunov D, Frost LS. F conjugation: back to the beginning. Plasmid. 2013;70(1):18-32.
20. Llosa M, Gomis-Rüth FX, Coll M, de la Cruz F. Bacterial conjugation: a two-step mechanism for DNA transport. Mol Microbiol. 2002;45(1):1-8.
21. Schroder G, Lanka E. The mating pair formation system of conjugative plasmids-A versatile secretion machinery for transfer of proteins and DNA. Plasmid. 2005;54(1):1-25.
22. Haft RJ, Mittler JE, Traxler B. Competition favours reduced cost of plasmids to host bacteria. ISME J. 2009;3(7):761-9.
23. Zatyka M, Thomas CM. Control of genes for conjugative transfer of plasmids and other mobile elements. FEMS Microbiol Rev. 1998;21:291-319.
24. Darphorn TS, Bel K, Koenders-van Sint Anneland BB, Brul S, Ter Kuile BH. Antibiotic resistance plasmid composition and architecture in Escherichia coli isolates from meat. Sci Rep. 2021;11(1):2136.
25. National Institute for Public Health and the Environment. Nethmap-Maran 2020. The Netherlands; 2020.
26. Schuurmans JM, Nuri Hayali AS, Koenders BB, ter Kuile BH. Variations in MIC value caused by differences in experimental protocol. J Microbiol Methods. 2009;79(1):44-7.
27. Garcia-Fernandez A, Fortini D, Veldman K, Mevius D, Carattoli A. Characterization of plasmids harbouring qnrS1, qnrB2 and qnrB19 genes in Salmonella. J Antimicrob Chemother. 2009;63(2):274-81.
28. Carattoli A, Zankari E, Garcia-Fernandez A, Voldby Larsen M, Lund O, Villa L, et al. In silico detection and typing of plasmids using PlasmidFinder and plasmid multilocus sequence typing. Antimicrob Agents Chemother. 2014;58(7):3895-903.
29. Bortolaia V, Kaas RS, Ruppe E, Roberts MC, Schwarz S, Cattoir V, et al. ResFinder 4.0 for predictions of phenotypes from genotypes. J Antimicrob Chemother. 2020.
30. Elowitz MB, Levine AJ, Siggia ED, Swain PS. Stochastic Gene Expression in a Single Cell. Science. 2002;297:1183-6.
31. Evans CGT, Herbert D, Tempest DW. Chapter XIII The Continuous Cultivation of Micro-organisms. Methods in Microbiology1970. p. 277-327.
32. Koraimann G, Wagner MA. Social behavior and decision making in bacterial conjugation. Front Cell Infect Microbiol. 2014;4:54.
33. Lundquist PD, Levin BR. Transitory depression and the maintenance of conjugative plasmids. Genetics. 1986;113:483-97.
34. Gama JA, Zilhao R, Dionisio F. Plasmid Interactions Can Improve Plasmid Persistence in Bacterial Populations. Front Microbiol. 2020;11:2033.
35. Benz F, Huisman JS, Bakkeren E, Herter JA, Stadler T, Ackermann M, et al. Plasmid- and strain-specific factors drive variation in ESBL-plasmid spread in vitro and in vivo. ISME J. 2021;15(3):862-78.
36. Pietsch M, Pfeifer Y, Fuchs S, Werner G. Genome-Based Analyses of Fitness Effects and Compensatory Changes Associated with Acquisition of bla CMY-, bla CTX-M-, and bla OXA-48/VIM-1-Containing Plasmids in Escherichia coli. Antibiotics (Basel). 2021;10(1).
37. Prensky H, Gomez-Simmonds A, Uhlemann AC, Lopatkin AJ. Conjugation dynamics depend on both the plasmid acquisition cost and the fitness cost. Mol Syst Biol. 2021;17(3):e9913.

38. Madsen JS, Burmolle M, Hansen LH, Sorensen SJ. The interconnection between biofilm formation and horizontal gene transfer. FEMS Immunol Med Microbiol. 2012;65(2):183-95.
39. Molin S, Tolker-Nielsen T. Gene transfer occurs with enhanced efficiency in biofilms and induces enhanced stabilisation of the biofilm structure. Current Opinion in Biotechnology. 2003;14(3):255-61.
40. Virolle C, Goldlust K, Djermoun S, Bigot S, Lesterlin C. Plasmid Transfer by Conjugation in Gram-Negative Bacteria: From the Cellular to the Community Level. Genes (Basel). 2020;11(11).
41. Garnett JA, Martinez-Santos VI, Saldana Z, Pape T, Hawthorne W, Chan J, et al. Structural insights into the biogenesis and biofilm formation by the Escherichia coli common pilus. Proc Natl Acad Sci U S A. 2012;109(10):3950-5.
42. Trunk T, Khalil HS, Leo JC. Bacterial autoaggregation. AIMS Microbiol. 2018;4(1):140-64.
43. May T, Okabe S. Escherichia coli harboring a natural IncF conjugative F plasmid develops complex mature biofilms by stimulating synthesis of colanic acid and Curli. J Bacteriol. 2008;190(22):7479-90.
44. Reisner A, Holler BM, Molin S, Zechner EL. Synergistic effects in mixed Escherichia coli biofilms: conjugative plasmid transfer drives biofilm expansion. J Bacteriol. 2006;188(10):3582-8.

Table 1. List of *E. coli* strains used as donors, names of recipient strains and their chromosomally encoded antibiotic resistance and characterization of plasmids as found within the donor strains, showing number of plasmids (#), replicon types and beta-lactamase genes. (Sequences can be found at Genbank accession numbers MW390511 to MW390552) .

Donor	Recipient		Donor plasmids		
Strain	Strain	resistance	#	Replicon type	Beta-lactamase
E. coli 2073	*E. coli* MG1655	chlor^R	2	IncI1, IncX4	$bla_{CTX-M-1}$
E. coli 2082	*E. coli* MG1655	chlor^R	5	IncI1, IncX4, IncX1, IncFIB/FII, p$_{phage}$	bla_{SHV-12}, bla_{TEM} (3x)
E. coli 3153	*E. coli* MG1655	chlor^R	3	IncI1, IncX4, IncFII	$bla_{CTX-M-1}$
E. coli 3156	*E. coli* MG1655	chlor^R	3	IncB/0/K/Z, IncX4, IncFIB/FII	bla_{CMY-2}, bla_{TEM-1B}
E. coli 3170	*E. coli* MG1655	chlor^R	3	IncI1, IncFIB, IncFIC/FII	$bla_{CTX-M-1}$
	P. aeruginosa POA-1				
	P. aeruginosa PA-1				
	E. faecalis OG1RF				
E. coli 3171	*E. coli* MG1655	chlor^R	2	IncI1, IncFII	bla_{CMY-2}
E. coli 3182	*E. coli* MG1655	chlor^R	3	IncN, IncFII, IncFIB	$bla_{CTX-M-55}$, bla_{TEM-1B}
E. coli 3203	*E. coli* MG1655	chlor^R	2	IncI, IncFII	$bla_{CTX-M-8}$
E. coli 3215	*E. coli* MG1655	chlor^R	4	IncI2, IncX4, IncFIB/FIC, p$_{phage}$	$bla_{TEM-52c}$
E. coli 3227	*E. coli* MG1655	chlor^R	2	IncI1, IncFIB/FII	$bla_{CTX-M-2}$, bla_{TEM-1B}
E. coli 3231	*E. coli* MG1655	chlor^R	3	IncN, IncFIB/FII, p0111	bla_{TEM}, bla_{SHV-12}
E. coli 3277	*E. coli* MG1655	enr^R	1	IncFIB/FIC	$bla_{CTX-M-55}$
E. coli 3284	*E. coli* MG1655	enr^R	1	IncFIB/FII	bla_{CMY-2}*
E. coli 3301	*E. coli* MG1655	chlor^R	3	IncI1, IncX1, IncFIB/FII	$bla_{CTX-M-1}$
E. coli 3308	*E. coli* MG1655	enr^R	4	IncI1, IncY, IncFIB, IncFIC/FII	$bla_{CTX-M-1}$
E. coli 3310	*E. coli* MG1655	chlor^R	2	IncB/0/K/Z, IncFIB/FIV	bla_{CMY-2}
E. coli 3334	*E. coli* MG1655	chlor^R	2	IncX1, col156	$bla_{CTX-M-32}$
E. coli MG1655 pIncI3170	*E. coli* JW3686	kan^R	1	IncI1	$bla_{CTX-M-1}$
E. coli JW3686 pIncI3170	*E. coli* MG1655	chlor^R	1	IncI1	$bla_{CTX-M-1}$

*Beta-lactamase is unconfirmed if located in the plasmid or the chromosome

Table 2. Number of transconjugants found for transfer with *E. coli* ESBL 3170 as donor and *E. faecalis* OG1RF and two *P. aeruginosa* strains as recipient.

Species	Transconjugants/million cells
E. faecalis OG1RF	0
P. aeruginosa PAO-1	0
P. aeruginosa PA-1	0

(This table can be omitted without losing information, but will be useful for a reader who only glances at the article instead of reading it thoroughly.)

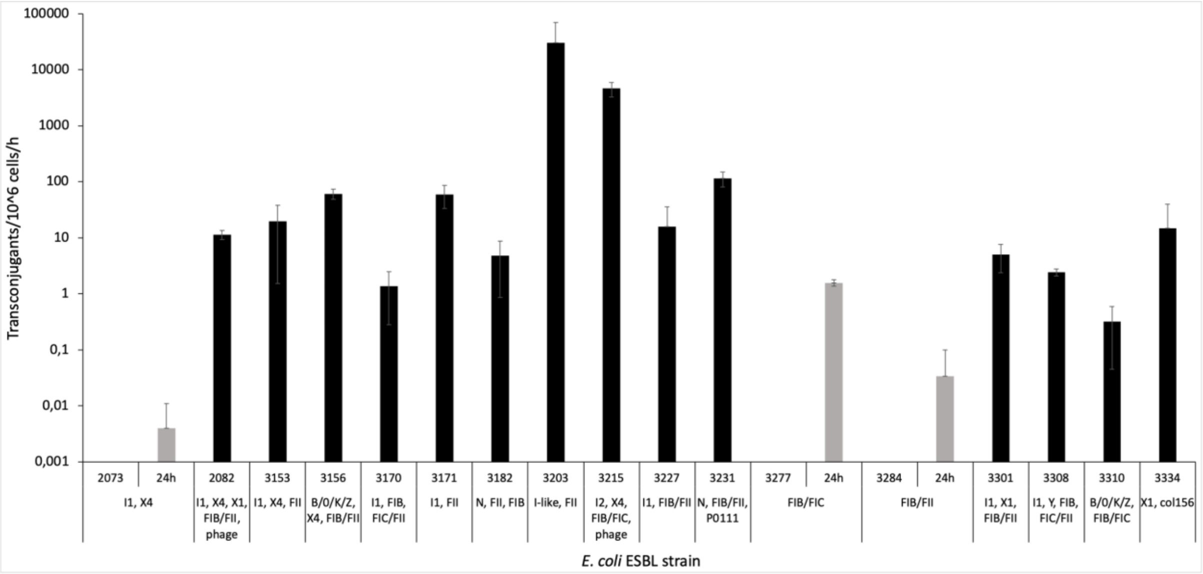

Figure 1. Transconjugants/10^6 cells/hour per strain in log scale with standard deviation and the corresponding plasmid types as present in the donor strain. All measurements are as shown after 1 hour. When no transconjugants were obtained after 1 hour, the 24-hour sample is also shown.

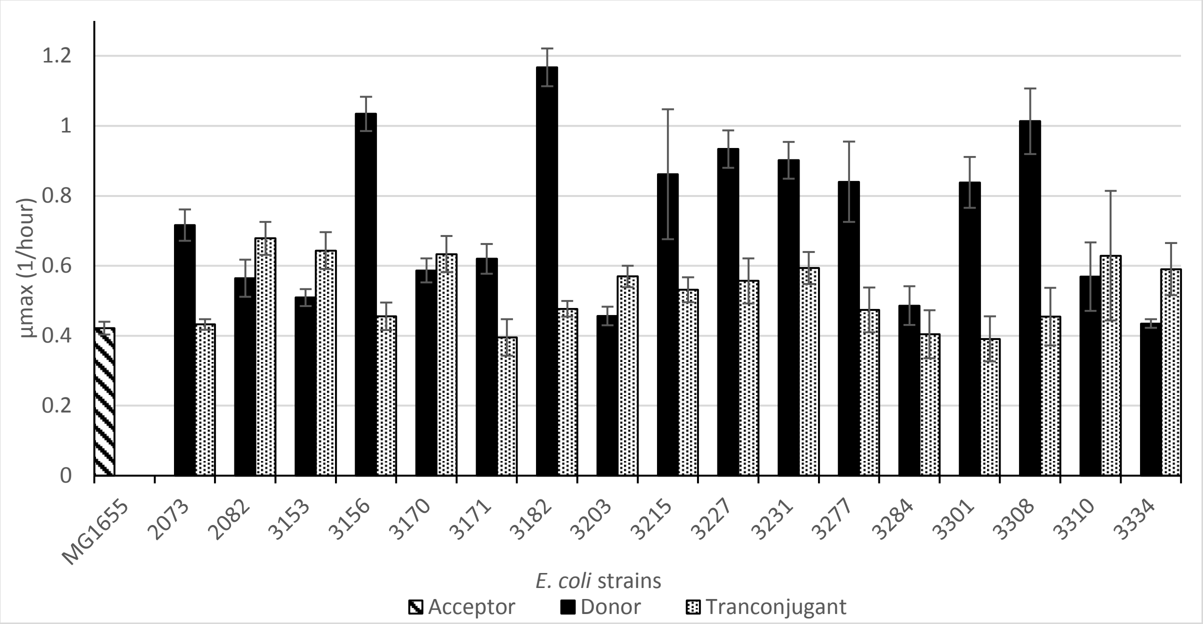

Figure 2. average μ_{max} (h^{-1}) per recipient (diagonal stripes), donor (solid) and transconjugant (dots) strain with standard deviation. Average values were determined from at least 2 replicates, that individually were measured 8 times.

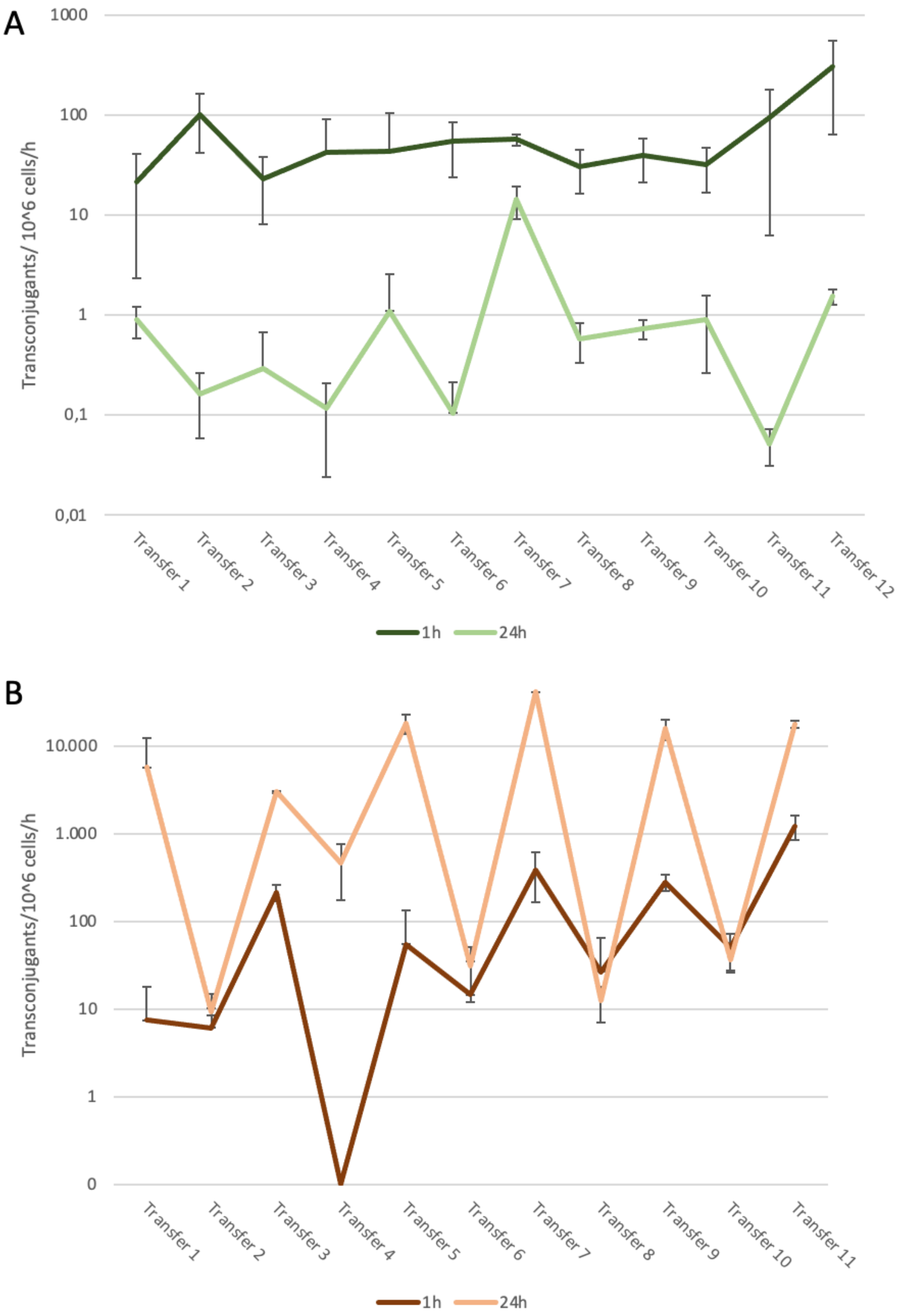

Figure 3. Transconjugants per million cells per hour found after multiple transfers with standard method of transfer(**A**) or addition of glucose (**B**) measured after 1 and 24 hours. Two recipient s were used: *E. coli* JW3686 with kanamycin resistance (uneven transfers) and *E. coli* MG1655 with chloramphenicol resistance (even transfers). The obtained transconjugants were used as a donor

strain for the next transfer round. Standard deviations are shown but are distorted because of the use of the log scale.

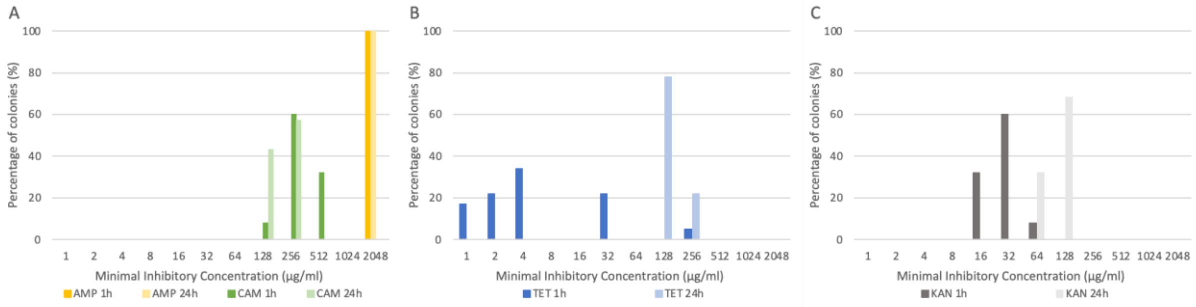

Figure 4. The percentage of isolated colonies found with a specific minimal inhibitory concentration (MIC) for antimicrobials: ampicillin and chloramphenicol (**A**), tetracycline (**B**) and kanamycin (**C**) after co-incubation of *E. coli* MG1655 and *E. coli* ESBL2082 for 1 hour or 24 hours and plating on double selective plates with ampicillin and chloramphenicol.

Figure 5 added in separate file

Figure 5. TEM micrographs taken from *E. coli* species ESBL2073 PFA fixed (**A**), ESBL3153 (**B**), MG1655 (**C**), Indole knockout (**D**) and E the mixture of ESBL3153 together with MG1655. In right column the close-up of pili (arrowheads) or flagellum (arrows) of same cells as in A-E.

Figure

Click here to access/download;Figure;Darphorn et al

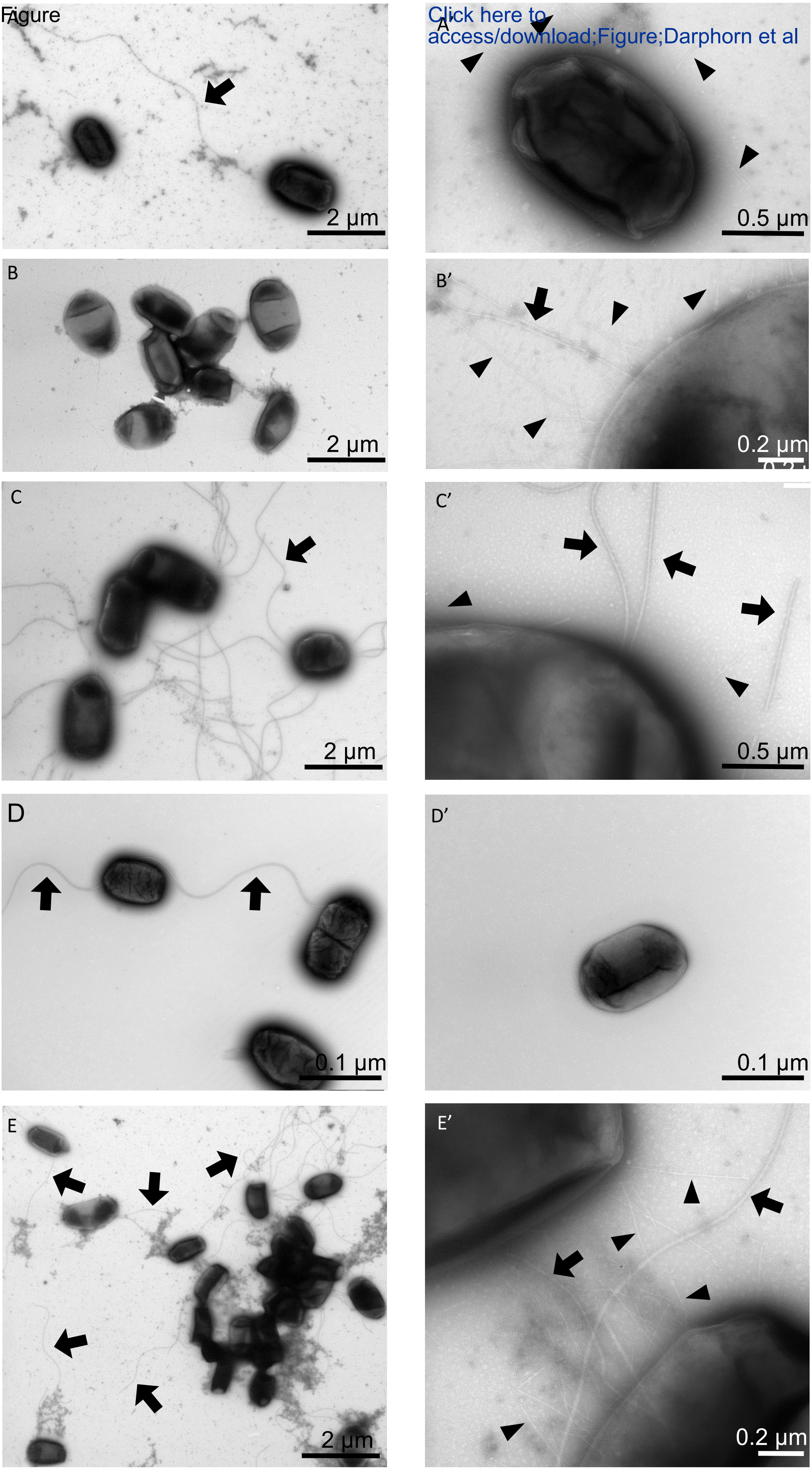

Supplement: Attachment — Submitted filename: HUD PONE-D-21-39054_reviewer.pdf [file pone.0270205.s001.pdf]
