# Supplementary material for: Transfer dynamics of multi-resistance plasmids in Escherichia coli isolated from meat
Source: PLoS One. 2022 Jul 7;17(7):e0270205. doi: 10.1371/journal.pone.0270205 (PMC9262221; doi:10.1371/journal.pone.0270205)
Supplement: Attachment — Submitted filename: Letter of rebuttal Darphorn et al.,.docx [file pone.0270205.s002.docx]

Response to reviewers HUD-PONE-D-21-39054; Darphorn et al.,

To: Dr. H.U. Dahms
Academic Editor, PLOS ONE

Amsterdam, February 14^th^, 2022

Dear Dr. Dahms,

Herewith you find attached the revision of our manuscript “Transfer dynamics of multi-resistance plasmids in *Escherichia coli* isolated from meat”.

We would like to express our gratitude to the reviewer whose suggestions clearly improved the text. We admire her/his eye for detail and have incorporated almost all editorial suggestions. The 3 exceptions are detailed below, as are the 2 other points made by the reviewer. As the suggestions were so clear and could all be incorporated in a straightforward manner we do not provide a marked up text.

Changes to be discussed:

Line 63: “enables” refers to “machinery” not to “plasmids”

Line 145: accepted and corrected accordingly. NB: after this correction all line numbers shift by 1.

Line 229 (230) and line 257 (258): “suggests” is preferable over “suggested” because it is a conclusion that is not limited in time.

Line 274: The flagella were visible in the EM pictures, but obviously not involved in plasmid transfer. We eliminated the mention of flagella as this was only confusing the issue.

Line 296 (297): In our opinion this suggestion is not an improvement.

All other suggestions are incorporated in the new text.

We hope the revised manuscript can be accepted for publication in PLOS-ONE.

Sincerely,

Benno ter Kuile
