# Supplementary material for: Transfer dynamics of multi-resistance plasmids in Escherichia coli isolated from meat
Source: PLoS One. 2022 Jul 7;17(7):e0270205. doi: 10.1371/journal.pone.0270205 (PMC9262221; doi:10.1371/journal.pone.0270205)
Supplement: Attachment — Submitted filename: REVIEW -Transfer dynamics of multiresistance plasmids in E.coli (1).docx [file pone.0270205.s003.docx]

Transfer dynamics of multi-resistance plasmids in Escherichia coli isolated from meat

The idea of this research work is significant in the context of effect of the presence of multiple plasmids in *E.coli* in transferring resistance to recipient species via conjugation process. The overall test results done are effective and various parameters have been addressed with a clear representation of the result. Appropriate concentrations of antibiotics used, OD values to be considered are clearly mentioned which can be effectively used as reference by other authors, universities and researchers. The provision of GenBank accession numbers of various strains adds more value to the text. Another important aspect that needs to be appreciated is the confirmation of the species specific nature of plasmid transfer.

In the methods section, there is more need to focus on the method of isolation of resistant *E.coli* strains from meat. Why only chicken, turkey and bovine was used for plasmid isolation and why not mice (for example) or any other species? Another point that needs to be addressed is about the co-incubation time as to why it has been conducted at only 1 hour and another for 24 hours. Why haven’t the authors tried to conduct for other time periods or gradient time interval range, or if they have conducted, then it should be mentioned in the methods section.

The size of the font set for figures 1 and 4 can be enlarged to an extent when compared with figures 3 and 4.

In line 52, ‘Resistance plasmids play a crucial **rule**’ should be changed as ‘Resistance plasmids play a crucial **role**’

In line 102, ‘ResFinder to determine and confirm and find’ could be changed to ‘to determine, confirm and find’.

In line 179, ‘Before plating the **transformation** on selective plates’ is suggested for change as ‘before plating the **transformed cells** on selective plates’

Check on the overall grammar and flow of the content.

Otherwise, the article is overall well written and explained and can be advised for acceptance with minor corrections.
